# AMiCUS—A Head Motion-Based Interface for Control of an Assistive Robot

**DOI:** 10.3390/s19122836

**Published:** 2019-06-25

**Authors:** Nina Rudigkeit, Marion Gebhard

**Affiliations:** Group of Sensors and Actuators, Department of Electrical Engineering and Applied Physics, Westphalian University of Applied Sciences, 45877 Gelsenkirchen, Germany; marion.gebhard@w-hs.de

**Keywords:** assistive technology, human-machine interaction, motion sensors, robot control, tetraplegia, IMU, AHRS, head control, gesture recognition, real-time control

## Abstract

Within this work we present AMiCUS, a Human-Robot Interface that enables tetraplegics to control a multi-degree of freedom robot arm in real-time using solely head motion, empowering them to perform simple manipulation tasks independently. The article describes the hardware, software and signal processing of AMiCUS and presents the results of a volunteer study with 13 able-bodied subjects and 6 tetraplegics with severe head motion limitations. As part of the study, the subjects performed two different pick-and-place tasks. The usability was assessed with a questionnaire. The overall performance and the main control elements were evaluated with objective measures such as completion rate and interaction time. The results show that the mapping of head motion onto robot motion is intuitive and the given feedback is useful, enabling smooth, precise and efficient robot control and resulting in high user-acceptance. Furthermore, it could be demonstrated that the robot did not move unintendedly, giving a positive prognosis for safety requirements in the framework of a certification of a product prototype. On top of that, AMiCUS enabled every subject to control the robot arm, independent of prior experience and degree of head motion limitation, making the system available for a wide range of motion impaired users.

## 1. Introduction

Tetraplegia is defined as the partial or total loss of motor and/or sensory function of the arms, legs, trunk and pelvic organs due to damage of the cervical segments of the spinal cord [1]. Besides traumatic injuries also disorders like cerebral palsy, amyotrophic lateral sclerosis or multiple sclerosis can lead to this severe disability [2]. The worldwide incidence of tetraplegia is estimated to lie between 3.5 and 27.7 per million inhabitants per year. The percentage of tetraplegics of all cases of spinal cord injuries increased within the last decades [3].

Tetraplegic patients require extensive home care services and often retire from working life because they no longer meet the physical conditions. The development of assistive robots for the people concerned is of major importance to at least partly restore their autonomy, substantially improving their quality of life.

Different uni- or multimodal Human–Machine Interface (HMI) concepts for tetraplegics have already been tested. These interfaces consider the user’s remaining capabilities to voluntarily produce input signals, such as movement of eyes [4], head [5] or tongue [6], speech [7], breath [8], brain activity [9] or voluntary muscle contraction of neck [10], facial [11] or ear muscles [12]. The choice of the most suitable input modality depends on the preferences and physical abilities of each user as well as on the underlying control scheme. However, the goal of the research presented here was developing a solely head motion-based interface for real-time control of a robotic arm.

Common sensing modalities for head motion are ultrasound modules [13], ordinary cameras [14], infrared cameras, chin joysticks [15] and motion sensors [16]. In recent years, state-of-the-art motion sensors, such as Attitude Heading Reference Systems (AHRS) based on Micro Electro-Mechanical Systems (MEMS) technology, have gained increasing interest because they enable accurate motion measurement while being small, low-cost, lightweight, energy-efficient and self-contained, making them ideal for use in HMIs. For this reason, a MEMS AHRS has been considered the preferred choice to measure head motion within this work.

The majority of existing motion sensor-based head-controlled interfaces are limited to 2D-applications, such as control of mouse cursors [16,17,18,19,20], wheelchairs [17,21] or other vehicles [5]. Few attention has been paid to more complex applications, such as robot arm control. However, Williams et al. [22] use head orientation to control the Tool Center Point (TCP) of a robot arm. Furthermore, Fall et al. [23] use motion of the shoulders and neck to control the commercially available robot arm JACO [24]. The user can choose between different control modes of JACO to perform 3D-translations, arm and wrist rotations or control the fingers’ positions, respectively. The Human–Robot Interface (HRI) presented by Fall et al. requires additional switches to switch between these modes, though.

In our research group, we have developed the AMiCUS system, which is the first interface that uses only head motion to produce all the necessary signals and commands for real-time control of an application with more than three Degrees of Freedom (DOF), such as a robotic arm. Some of the major criteria for the development of such an HRI are the following:

1. The HRI should be adaptive, always using the full available neck range of motion of the user.
2. The relationship between performed head motion and resulting robot motion has to be intuitive.
3. The HRI must reliably distinguish between unintended head motion, head motion intended for direct control and head motion to generate switching commands.
4. The HRI has to give sufficient and useful feedback to the user to allow safe and efficient operation.
5. The HRI must enable the user to perform smooth, precise and efficient robot movements in Cartesian space.
6. The user should enjoy using the HRI.

AMiCUS has been designed with special attention to these requirements. A tetraplegic with severe head motion limitation has been involved in the whole development cycle to ensure the system’s relevance for the target group.

In the next section, the hardware, software and signal processing of the resulting system are described. Afterwards, the experimental setup of a user study with 13 able-bodied and 6 tetraplegic subjects to validate the system is presented. Subsequently, the results are presented and discussed. In the last section, these results are compared against aforementioned criteria for a head motion-based HRI.

## 2. AMiCUS

Within this section we introduce AMiCUS. AMiCUS stands for Adaptive Head Motion Control for User-friendly Support. The demonstrator AMiCUS is the result of several years of research within our research group. First, we will give an overview of our research activities. Then, we describe the sensor placement. This is followed by a description of the used hardware. Next, we present how robot groups have been built in order to control all DOFs of the robot with head motion. Afterwards, the two modes of AMiCUS, namely Robot Control Mode and Cursor Control Mode, are described in detail. The section closes with the presentation of the necessary calibration routines.

### 2.1. Relation to Previously Published Work

In Reference [25] suitable control modes for real-time control using head motion have been analyzed. Based on the results, a first iteration of the general control structure has been evaluated in References [26,27]. An algorithm to detect head gestures, which can be used as switching commands as part of the chosen control structure has been presented in Reference [28]. Within this work, the whole system that resulted from all previous research is presented and validated in a user study. A usability study of an alternative control structure and GUI for the AMiCUS system has been presented by Jackowski et al. [29]. The system in this publication uses all four head gestures defined in Reference [28] to switch between robot groups, whereas the interface described here uses a control structure based on the work published in References [26,27]. The main advantage of the version presented by Jackowski et al. is that switching between groups is faster, while the main advantage of the version presented here is that it can be used by a wider range of users as explained in detail later in this work.

### 2.2. Sensor Placement

A MEMS AHRS was chosen to measure the user’s head motion. A typical AHRS outputs raw sensor data from a 3D accelerometer, a 3D gyroscope and a 3D magnetometer, as well as sensor orientation obtained from such raw data.

**Figure 1:**
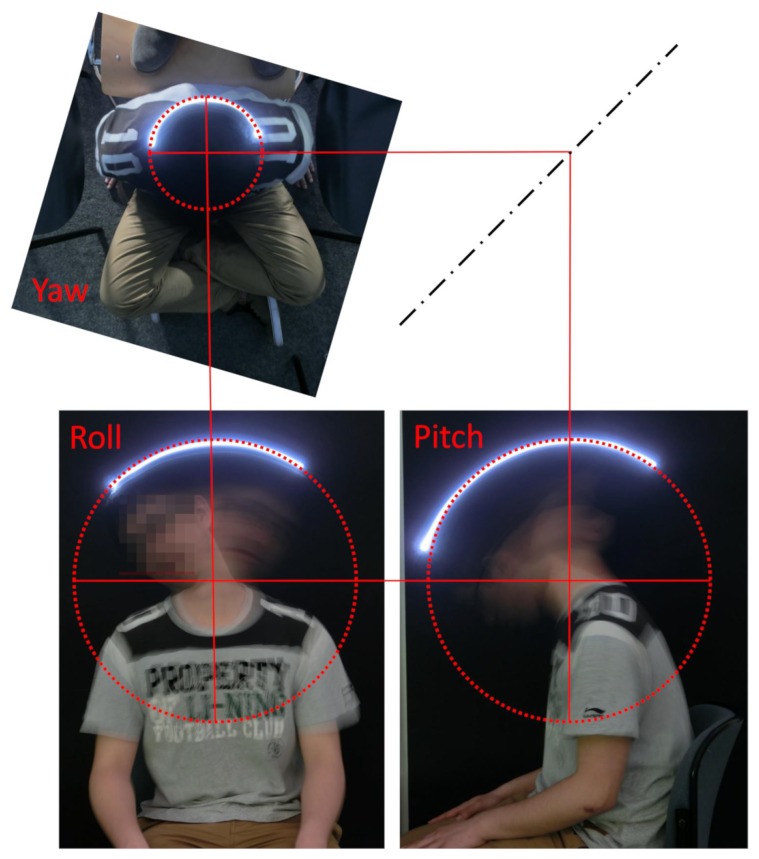
Kinematics of the cervical spine. From the kinematic point of view, the human cervical spine can be approximated by a ball joint. That means, every motion can be divided into single rotations around three orthogonal axes that intersect in one point. This point, that is, the center of rotation, roughly coincides with the location of the thyroid gland. As a result, a rigid body placed onto a human head moves on a spherical surface during head motion.

In Reference [30] we showed that a rigid body placed onto a human head is moved on a spherical surface during head motion (Figure 1). For the sake of simplicity, the sensor placement was chosen in a way that the sensor yaw axis and the approximated yaw axis of the user’s cervical spine coincided. Given this sensor placement, changes in head and sensor orientation are identical and a transformation of sensor orientation to head orientation is not needed. Nonetheless, an offset calibration remains necessary to define the zero position of the user’s head. Every head motion apart from rotation around the yaw-axis results in additional linear sensor movement as the sensor is not rotated around its own center.

### 2.3. Hardware

**Figure 2:**
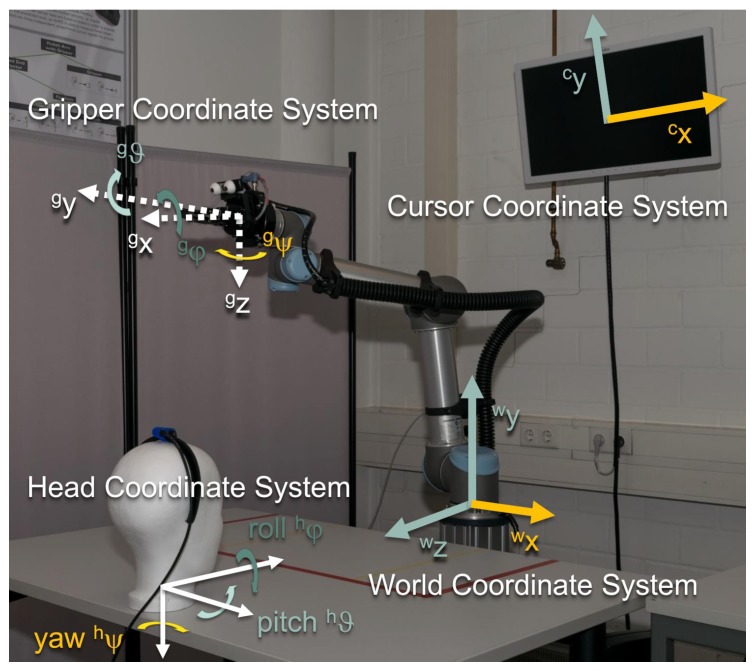
Coordinate systems of the AMiCUS system. Degrees of Freedom (DOFs) of the same color are controlled by the same head DOF. The zero orientation of the head coordinate system depends on whether the cursor or the robot is controlled. During robot control, the head coordinate system is denoted by ${}^{h_{r}}\alpha ={\;}^{h_{r}}\left(\varphi ,\vartheta ,\psi \right){}^{T}$ and ${}^{h_{c}}\alpha ={\;}^{h_{c}}\left(\varphi ,\vartheta ,\psi \right){}^{T}$ during cursor control.

AMiCUS detects head motion using a Hillcrest FSM-9 motion sensor system [31], which is placed as described in the previous section. Output data of the sensor system under static conditions and during head motion are provided in the Supplementary Materials. The acquired sensor data is processed on a desktop computer with the AMiCUS software, which is written in C++ using the Qt framework [32]. The resulting control signals are transmitted to a Universal Robots UR5 robot arm [33] which is equipped with a Robotiq 2-Finger parallel gripper [34]. By default, the position of the gripper can be controlled in world coordinates ${}^{w}p={}^{w}\left(x,y,z\right){}^{T}$ as well as gripper coordinates ${}^{g}p={}^{g}\left(x,y,z\right){}^{T}$ using inverse kinematics. These coordinate systems are shown in Figure 2. Rotations of the gripper are performed in gripper coordinates ${}^{g}\alpha ={}^{g}\left(\varphi ,\vartheta ,\psi \right){}^{T}$. The gripper can be opened and closed in order to interact with objects. The position of the gripper’s fingers is denoted as β. All these DOFs, ${}^{r}s=\left({}^{w/g}p,{}^{g}\alpha ,\beta \right){}^{T}$, can be controlled proportionally. That means that the signal amplitude can be varied continuously. A Logitech C930e webcam [35] is mounted on the gripper to provide feedback during positioning and gripping. The camera image is part of the Graphical User Interface (GUI) of AMiCUS. The GUI is displayed on an ordinary computer screen in front of the user.

### 2.4. Robot Groups

**Figure 3:**
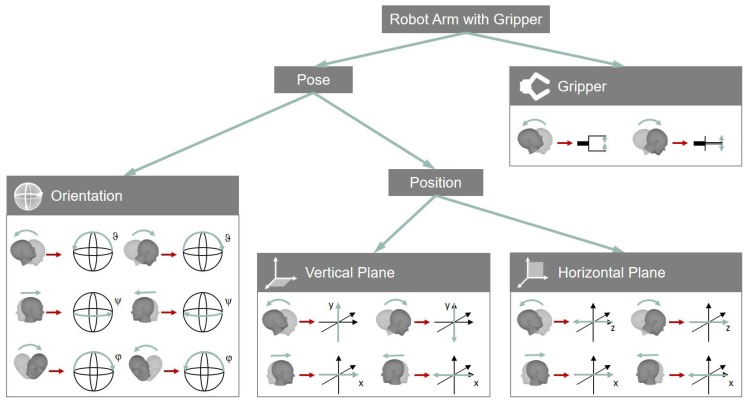
Mapping of head DOFs onto robot DOFs. Four different groups, that is, Gripper, Orientation, Vertical Plane and Horizontal Plane, are depicted. The user is able to switch between groups in order to control all DOFs of the robot.

Three proportional control signals are provided through head motion, that is, ${}^{h}\alpha ={\;}^{h}\left(\varphi ,\vartheta ,\psi \right){}^{T}$. With these signals, seven DOFs of the robot, ${}^{r}s$, have to be controlled. That means, direct robot control in terms of a 1:1-mapping is not feasible. For this reason, groups containing maximum three DOFs of the robot have been defined, namely Gripper, Vertical Plane, Horizontal Plane and Orientation. The DOFs of the head have been mapped onto the DOFs of the robot as follows (Figure 3):

In the Gripper group, the gripper can be opened and closed using the pitch motion of the user’s head (${}^{h}\vartheta \mapsto \beta$). The group Vertical Plane enables the user to move the gripper within a plane that is perpendicular to the user’s line of sight. The user can move the gripper to the left or right by turning the head to the left or right (${}^{h}\psi \mapsto {}^{w}x$). In order to move the gripper up or down, one has to tilt the head up or down (${}^{h}\vartheta \mapsto {}^{w}y$). The gripper can be moved back or forth in the Horizontal Plane group by tilting the head down or up (${}^{h}\vartheta \mapsto {}^{w}z$). Additionally, the user can move the robot left and right the same way as in the Vertical Plane group.

The orientation of the gripper is controlled in the Orientation group. Each rotary movement of the head is mapped onto the corresponding rotation of the gripper. That means, the roll rotation of the gripper is controlled by the roll rotation of the head (${}^{h}\varphi \mapsto {}^{g}\varphi$); the control of the pitch rotation of the gripper is performed using the pitch rotation of the head (${}^{h}\vartheta \mapsto {}^{g}\vartheta$); and the yaw rotation of the gripper is controlled by the yaw rotation of the head (${}^{h}\psi \mapsto {}^{g}\psi$).

### 2.5. Robot Control Mode

During direct robot control within one of the robot groups, the position along a head DOF is projected onto the velocity along a robot DOF $\left(\gamma \mapsto \dot{g}\right)$, with $\gamma \in {}^{h}\alpha$ and $g\in {}^{r}s$ [25].

**Figure 4:**
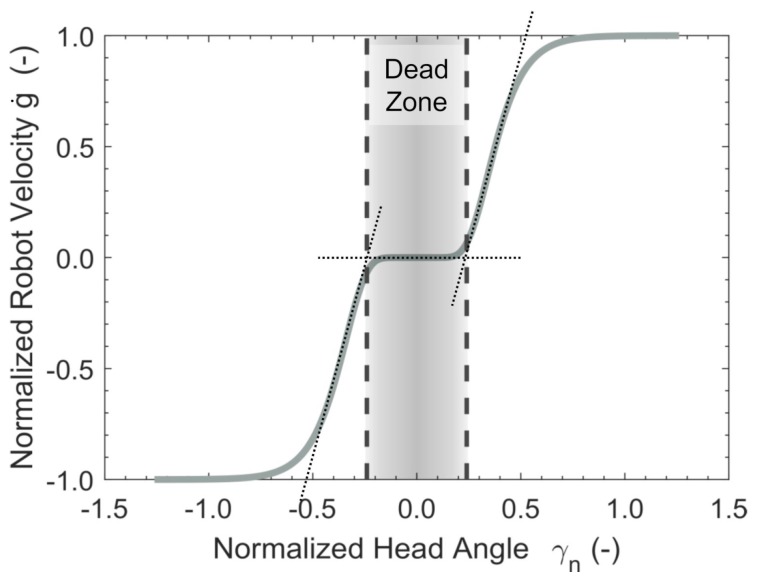
Robot control transfer function. A Gompertz-function is used as transfer function. The used parameters are $A_{\max}=1$, δ=−30 and r=−10. The space between the dashed lines indicates the deadzone.

A Gompertz-function is used as the transfer function (Figure 4). Using this function, head motion close to zero position, which is likely to be unintended, is not translated into robot motion (Deadzone). If the user keeps increasing head deflection, the robot slowly starts to move into the desired direction. Small deflection angles of the head enable slow, precise robot control. An increase of head deflection leads to an exceedingly smooth transition to fast, imprecise robot control. Mathmatically, the implemented transfer function can be formulated as:

(1a) $$\begin{array}{ccc} \dot{g} & = & \left\{\begin{array}{cc} A_{\max}\;\cdot \;e^{\delta \;\cdot \;e^{r\;\cdot \;\gamma_{n}}} & \mathrm{if}\;\gamma >0\\ -A_{\max}\;\cdot \;e^{\delta \;\cdot \;e^{-r\;\cdot \;\gamma_{n}}} & \mathrm{else} \end{array}\right. \end{array}$$

(1b) $$\begin{array}{ccc} \gamma_{n} & = & \frac{\gamma }{\gamma_{\mathrm{th}}} \end{array}$$

The parameter $A_{\max}\in R^{+}$ indicates the upper asymptote. This corresponds to maximum velocity in *g*-direction. The coefficient $\delta \in R^{-}$ sets the displacements along the γ-axis and $r\in R^{-}$ indicates the growth rate. The parameters δ and *r* are identical for all the robot DOFs. The amplitude $A_{\max}$ is adjusted separately for the linear DOFs ${}^{w}p$, the rotational DOFs ${}^{g}\alpha$ and the gripper β. The parameter $\gamma_{\mathrm{th}}$ defines the range of motion along the γ-axis, which shall be used for control. This range as well as the zero position are obtained during a calibration routine as described later.

For every time step of length Δt, the new robot state ${}^{r}s{}_{\mathrm{new}}$ is computed using the following relationship:

(2) $$\begin{array}{c} {}^{r}s{}_{\mathrm{new}}={}^{r}s{}_{\mathrm{old}}+{}^{r}\dot{s}\;\cdot \;\Delta t \end{array}$$

If the linear acceleration of the sensor during control exceeds a certain threshold, robot control is deactivated. This is supposed to guarantee operational safety as quick movements are likely to not be intended for robot control. After deactiviation, the user has to turn his head to the zero position in order to continue control. This event is accompanied by visual and acoustic feedback.

**Figure 5:**
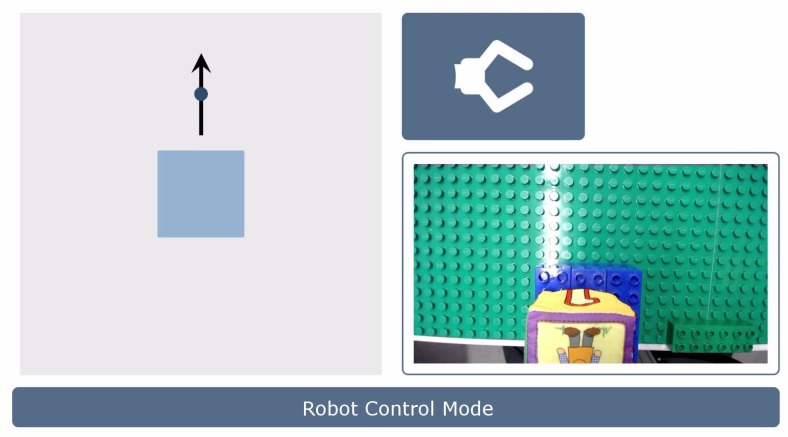
Graphical User Interface during Robot Control Mode. The GUI displays an icon of the current robot group (top right), the image of the gripper camera (bottom right), an information line (bottom) and feedback about the current head angle given by the arrow (left). The square represents the deadzone in which the robot arm cannot be moved.

The normalized head angles $\gamma_{n}$ are displayed in real-time on the GUI during Robot Control Mode. Additionally, a picture of the current robot group and the image of the gripper camera are shown (Figure 5).

During Robot Control Mode, all DOFs of the chosen group can be controlled simultaneously. However, switching is necessary to control the DOFs of another group. The user can leave the current group by performing a Head Gesture, which is described in the following.

#### Head Gesture

**Figure 6:**
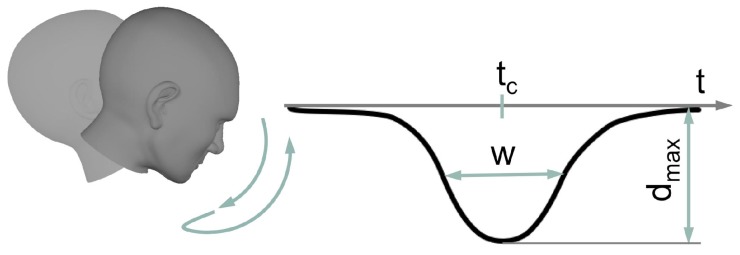
Head Gesture. The gesture is displayed with its ${}^{h_{r}}\vartheta$-angles over time *t*. Parameters: $d_{\max}$ = amplitude, *w* = peak width, $t_{c}$ = location of the peak center.

The Head Gesture denotes a nodding-like movement (Figure 6). More precisely, the user has to quickly move the head down starting from zero position and back. Mathematically, the shape of the gesture can be described by a Gaussian function:

(3) $$\begin{array}{c} d=d_{max}\;\cdot \;e^{-{\left(\frac{t-t_{c}}{w}\right)}^{2}} \end{array}$$

Maximum head displacement is expressed by the amplitude $d_{max}$, $t_{c}$ is the centroid and *w* is related to the peak width. The implemented algorithm for robust Head Gesture recognition in a real-time data stream has been presented in Reference [28]. Briefly summarized, the algorithm assumes that activity is present when the magnitude of linear acceleration of the sensor exceeds a certain threshold. If activity is present, both head angles ${}^{h_{r}}\alpha$ and sensor data are recorded. Recording stops when activity is no longer present. If the length of the recorded data lies between 0.25 s and 2 s and the dominating initial linear acceleration has been in positive ${}^{h}x$-direction (Figure 2), the recorded data might contain a Head Gesture. For validation, a Gaussian function is fitted to the recorded ${}^{h_{r}}\vartheta$-angles. The Head Gesture is classified if the following conditions are fulfilled:

- The neck was flexed sufficiently ($d_{\max}<$ −20${}^{\circ }$).
- The gesture was performed quickly enough (w< 0.4 s).
- The gesture was suffiently Gaussian-shaped ($R^{2}>0.75$).
- The maximum ${}^{h_{r}}\varphi$- and ${}^{h_{r}}\psi$-angles did not exceed 80% of $\left|d_{\max}\right|$

In case the Head Gesture has not been performed correctly, visual feedback is shown on the GUI that informs the user how to adjust a movement for successful Head Gesture detection. In case of correct gesture execution, the system switches from Robot Control Mode to Cursor Control Mode.

### 2.6. Cursor Control Mode

During Cursor Control Mode, the user controls a mouse cursor on the GUI using head motion to select a different robot group or to perform any other action, such as starting a calibration routine or pausing control.

To control the mouse cursor, the user’s head orientation is directly mapped onto the position of the cursor [25]. That means, the pitch-DOF is mapped linearly onto the ${}^{c}y$-axis of the screen (${}^{h}\vartheta \mapsto {}^{c}y$), while the yaw-DOF is mapped onto the ${}^{c}x$-axis of the screen (${}^{h}\psi \mapsto {}^{c}x$). This relationship is described by:

(4) cs=cxcy=diagmchc·hψhϑ+cb

The parameters mchc=mx,mychcT reflect the sensitivity. The parameters ${}^{c}b={}^{c}\left(b_{x},b_{y}\right){}^{T}$ indicate the cursor coordinates when the head is in its zero position, ${}^{h_{c}}\left(\vartheta ,\psi \right){}_{0}^{T}$. The corresponding values are obtained during a later described calibration routine.

**Figure 7:**
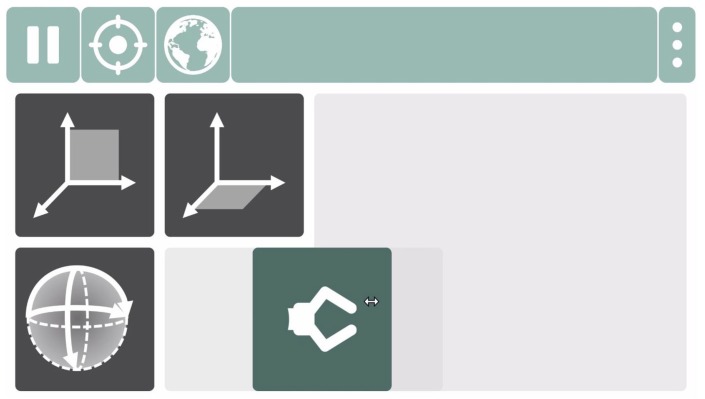
Graphical User Interface during Cursor Control Mode. The GUI contains one Slide Button for each robot group. The dwell buttons in the top toolbar allow the user to perform several actions, such as pausing control, starting calibration routines or exiting the program.

By moving the mouse cursor, the user interacts with the GUI that is shown during Cursor Control Mode (Cursor GUI, Figure 7). This GUI contains dwell buttons, for example, to start the calibration routines, pause control, switch between coordinate systems or close the program. A dwell button is activated by dwelling on it for 2 s. In addition to the dwell buttons, the GUI contains a Slide Button for each robot group. A robot group is selected by activating the corresponding Slide Button.

#### Slide Button

**Figure 8:**
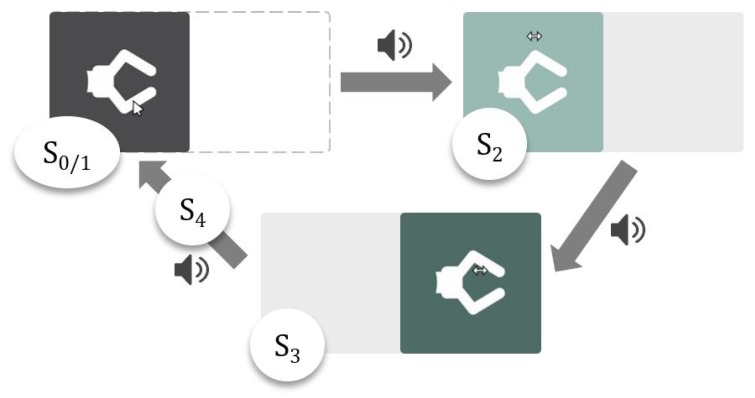
Slide Button. The following steps are necessary for successful activation: When the button is in its neutral state ($S_{0}$) the mouse cursor has to dwell in the button ($S_{1}$) until visual and acoustic feedback occurs. Then, the button has to be moved to the right along the rail ($S_{2}$). At the end of the rail visual and acoustic feedback is given. Next, the button has to be moved to the left along the rail ($S_{3}$). When the button reaches the initial position ($S_{4}$), it is activated and the assigned action is performed.

For successful activation of the Slide Button, the following steps are necessary (Figure 8): First, the user has to hover the Slide Button with the mouse cursor and dwell there for a certain time (State 1). Then, a rail rolls out in order to inform the user that the button can be slid to the right as indicated by the rail (State 2). If the cursor is moved to the right in a straight line, the Slide Button moves with it. If the user leaves the Slide Button area by moving the cursor up, down or left, the action is immediately aborted. After sliding the button to the right end of the rail (State 3), the user has to move the Slide Button back to the left. The action is aborted when leaving the Slide Button area to the top, bottom or right. If the Slide Button is moved to the left along the rail correctly, the rail rolls up and the robot group associated with the Slide Button is entered. Each state of the Slide Button is accompanied by visual and acoustic feedback.

### 2.7. Calibration Routines

#### 2.7.1. Robot Calibration

The calibration routine, which is needed to control the robot arm during Robot Control Mode, is called Robot Calibration. At the beginning of the Robot Calibration, the user has to define the zero position of the head coordinate system, ${}^{h_{r}}\alpha {}_{0}$. The zero position should be chosen in a way that the user faces the robot arm. This position has to be held for 2 s within a tolerance of 2${}^{\circ }$. Afterwards, the corresponding Euler angles of the sensor are saved as offset ${}^{s}\alpha {}_{0,r}={}^{s}\left(\varphi ,\vartheta ,\psi \right){}_{0,r}^{T}$. The offset is subtracted from the sensor angles ${}^{s}\alpha$ in order to obtain head angles ${}^{h_{r}}\alpha$.

After offset definition, the user has to perform each one repetition of neck flexion and extension, neck lateral bending to the left and to the right and neck rotation to the left and to the right. Pictures of these positions are displayed on the GUI. The users are instructed to move their heads as far as they can while still being able to see the robot. After holding a certain position, the corresponding head angle of the relevant DOF is saved and the next position is displayed. In this way two values are obtained for each DOF: One in positive direction ($\gamma_{+}$) and one in negative one ($\gamma_{-}$). To guarantee a symmetrical mapping, only the smaller value is used to define the range of motion $\gamma_{\mathrm{th}}$.

Whenever a calibration point is saved, acoustic feedback is given to the user. Furthermore, a status bar indicates how long the user has to hold the head stable until a calibration point is saved.

#### 2.7.2. Cursor Calibration

The calibration that is necessary to control the mouse cursor during Cursor Control Mode is denoted as Cursor Calibration. During Cursor Calibration, the user has to turn the head towards five targets, which are shown on the screen one after the other. A calibration point is saved when the user holds the head stable for 2 s within a tolerance of 2${}^{\circ }$. The first target is shown in the center of the screen. The user’s head orientation when facing this point is defined as the zero position of the head during cursor control, ${}^{h_{c}}\left(\vartheta ,\psi \right){}_{0}^{T}$. The corresponding sensor angles ${}^{s}\left(\vartheta ,\psi \right){}_{0,c}^{T}$ are defined as the offset between the head and the sensor coordinate system. For all samples, the offset is subtracted from the sensor angles ${}^{s}\left(\vartheta ,\psi \right){}^{T}$ to obtain the head coordinates ${}^{h_{c}}\left(\vartheta ,\psi \right){}^{T}$ explicitly. This step ensures that Gimbal Lock does not occur. For every target, the head angles ${}^{h_{c}}\left(\vartheta ,\psi \right){}^{T}$ and the position of the target on the screen ${}^{c}s$ are saved. The remaining targets are displayed in the upper left corner, center, lower right corner and in the center again. Whenever a calibration point is saved, acoustic feedback is given. At the end of the calibration procedure, the parameters mchc and ${}^{c}b$ are computed from the five calibration points using a minimum least squares fit. Head angles corresponding to the center of the screen are acquired three times to obtain more reliable data for this point. This is important, because the correct calibration of the center point has a major impact on the subjective calibration success perceived by the user.

## 3. Materials and Methods

The aim of the study was providing a proof-of-concept of the AMiCUS system. That means, showing that AMiCUS enabled people to perform simple manipulation tasks with the robot arm efficiently. Furthermore, we wanted to investigate if all aforementioned criteria for a head motion-based HRI were met and at which points AMiCUS could be developed further.

### 3.1. Subjects

Thirteen able-bodied subjects and six teraplegics took part in the experiments. The able-bodied subjects were recruited via announcements on the university website and in the local newspapers. Six of them were male, seven female. Their mean age was 37.0±15.0 years. The able-bodied subjects had no known neck motion limitations and carried out the experiments at the Westphalian University of Applied Sciences in Gelsenkirchen. The able-bodied subjects represented users with full Range of Motion (full ROM).

The tetraplegics were recruited via the BG-Hospital Hamburg where they also carried out the experiments. Five of them were male, one female. Their mean age was 35.7±15.2 years. The levels of injury ranged from C0 to C4. Subjects with both complete and incomplete injuries were included. The tetraplegics were chosen to have severe neck motion limitations in order to represent users with restricted Range of Motion (restricted ROM). It is worth noting that all of these tetraplegics were unable to operate the system presented in Reference [29] due to their neck motion limitations.

None of the subjects had prior experience with AMiCUS. The study was approved by the ethics committee of the University of Bremen and the subjects gave their informed consent prior to participation.

### 3.2. Experimental Setup

The subjects were seated in front of a table with an arrangement of platforms and softcubes according to the particular task to be performed. The robot arm was positioned on the opposite side of the table, facing the current subject.

The GUI was displayed on a 27″ screen, which was mounted behind the robot arm. The horizontal distance of the screen from the subject was approximately 2.4 m. The screen was positioned in a way that it was not occluded by the robot. Small deviations between the experimental setups for the able-bodied and tetraplegic subjects could not be avoided due to different spatial conditions.

The used sensor settings are shown in Table 1. The sampling rate for the raw and fused sensor data was set to 125 Hz. This is the recommended minimum sample rate for the sensor fusion algorithm to work properly. The accuracy of the orientation output was specified as 1.5${}^{\circ }$ by the manufacturer. The sensor data was downsampled to 60 Hz for further processing to save computing power while still displaying head orientation smoothly on the screen. The calculation of the joint angles using inverse kinematics and the physical robot movement needed almost 0.04 s. For this reason, the 60 Hz control signals to update the robot’s joint angles were further downsampled to 25 Hz.

Both the linear acceleration and angular position output were used for the gesture recognition. The data was processed as described in Reference [28]. The control signals for the cursor and robot arm were generated based on the angular position output as described in Section 2.

### 3.3. Procedure

A trial session, a predefined task and a complex task were part of the experimental study. The procedure was identical to the usability study performed with the alternative AMiCUS version as described in Reference [29]. This was done to allow for a better comparison of both versions. However, such a comparison is beyond the scope of the work presented here.

#### 3.3.1. Trial Session

Prior to the trial session, the subjects were shown an introduction video that demonstrated the basic working principle and modes of operation of AMiCUS. Video instructions were chosen to make sure every subject received the same information. After the video, the subjects were free to try the system for 10 min. They were encouraged to enter each robot group at least once.

#### 3.3.2. Predefined Task

**Figure 9:**
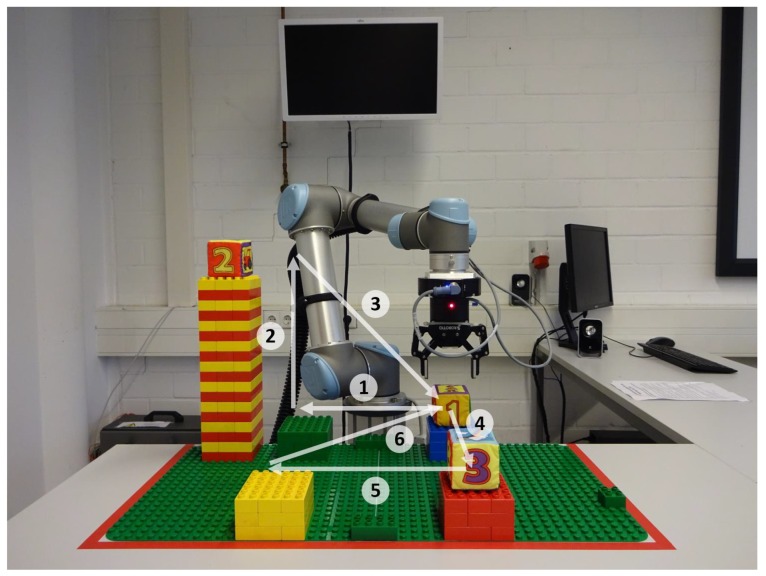
Experimental setup of the predefined task. The subjects were clearly instructed how to move the robot. Movements 1–3 had to be performed in the Vertical Plane group, movements 4–6 in the Horizontal Plane group. After movement 6, the subjects had to perform one 90${}^{\circ }$-rotation around each rotation axis.

After completing the trial session, a video of the predefined task was shown. For the tasks, five square-shaped platforms with 9.5 cm edge length and three softcubes with 6.5 cm edge length were arranged as shown in Figure 9.

First, cube 1 had to be moved from the blue to the green platform (1). After placing the cube on the green platform, the gripper had to be moved to the top of the tower (2) to grip cube 2 and move it to the blue platform (3). Afterwards, the gripper had to be moved to the red platform (4) and cube 3 had to be transported to the yellow platform (5). Finally, the gripper had to be moved to the blue platform (6). After arriving at the blue platform, cube 1 had to be gripped, lifted a little bit and rotated around 90${}^{\circ }$ in positive ${}^{g}\vartheta$-direction, then around 90${}^{\circ }$ in negative ${}^{g}\psi$-direction and finally around 90${}^{\circ }$ in positive ${}^{g}\varphi$-direction.

In order to solve this task, all subjects received detailed instructions about the desired control steps to be performed. Movements 1–3 had to be performed in the Vertical Plane group, movements 4–6 in the Horizontal Plane group and the rotations in the Orientation group.

#### 3.3.3. Complex Task

**Figure 10:**
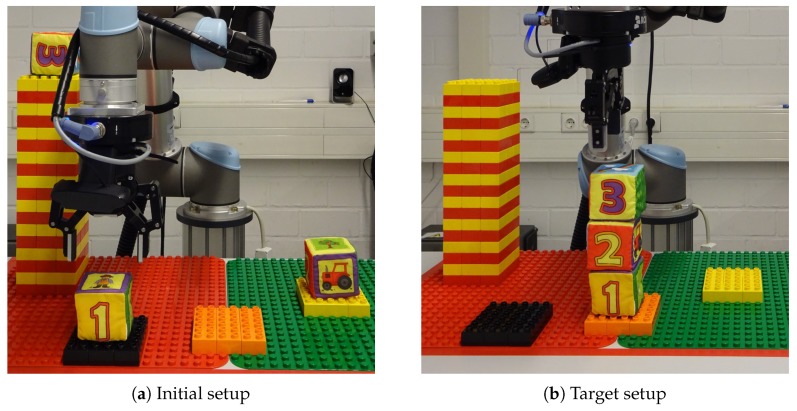
Experimental setup of the complex task. The users had to find their own control strategy to solve the task.

During the complex task, 3 softcubes were placed on a table as shown in Figure 10a. The users were told to stack the cubes on top of each other in a way that softcube 1 was on the formerly empty platform, softcube 2 in the middle and softcube 3 on top of the stack. In addition, a picture of the solution has been shown to the subjects prior to the task (Figure 10b). The subjects had to find their own control strategy to solve the task.

### 3.4. Evaluation Criteria

#### 3.4.1. Objective Evaluation

The completion rates were obtained for both the predefined and complex task. Subjects had only one trial to complete the tasks. A task was counted as failed when manual assistance from the experimenters was needed to proceed, when the subjects used their hands or when the task was executed incorrectly. Completion time was considered unsuitable as an evaluation criterion because of too many disturbing influences, for example, due to talking.

For both the Slide Button and the Head Gesture success rate and activation time were chosen as evaluation criteria. Success rate was evaluated using data of both the predefined and the complex task, whereas activation time contained only data from the predefined task. Activation time was defined as the time from the spoken command of the experimenter until the change of mode was visible on the GUI. That means that failed attempts were also included in the activation time. As a result, activation time and success rate are correlated. The medians of the activation times and success rates were first computed subject-wise. Based on these data, medians and quartiles were obtained for all subjects together. Differences between Slide Button and Head Gesture were evaluated utilizing paired-sample t-tests [36]. An overview of statistical tests can be found in Reference [37].

#### 3.4.2. Subjective Evaluation

For the subjective evaluation of the system, all subjects completed an evaluation sheet. This sheet contained 30 statements, regarding calibration, GUI, switching, mapping, transfer function and general aspects, which could be rated between 1 (*“I strongly disagree”*) and 5 (*“I strongly agree”*) (Table 2). Three additional statements regarding the speed of operation could be rated between 1 (*“Far too slow”*) and 5 (*“Far too fast”*). The answers were compared with each other using Friedman’s tests [38]. The significance level for the tests was 5%.

## 4. Results and Discussion

### 4.1. Objective Evaluation

#### 4.1.1. Completion Rates

All subjects, independent of available range of motion, were able to get a general understanding of the control structure and could move the robot arm in a controlled manner, as demonstrated in the trial session. It is worth mentioning that all subjects were first-time users of AMiCUS and did not have prior experience with any other head-controlled system.

**Figure 11:**
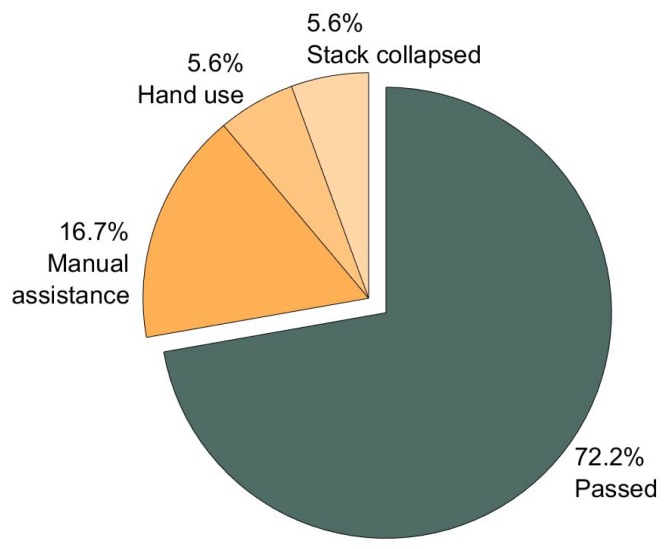
Completion rate of the complex task. There was no statistical difference between the users with full and restricted ROM. The overall completion rate of the complex task was 72.2% (n = 18).

The Fisher’s exact test [39] indicated that there was no significant difference between the users with full and restricted ROM regarding task completion rates. Therefore, the completion rates are presented for all subjects together. All subjects were able to complete the predefined task, resulting in a completion rate of 100%. The overall completion rate of the complex task was 72.2% (Figure 11). In 16.7% of the cases, manual assistance was required to solve the task at all or within a reasonable time period. In each 5.6% of the cases, hand-use or stack-collapse led to task failure. It is notable, that 60% of the non-completions resulted from inaccurate gripper positioning in ${}^{w}z$-direction.

#### 4.1.2. Success Rates and Activation Times

**Figure 12:**
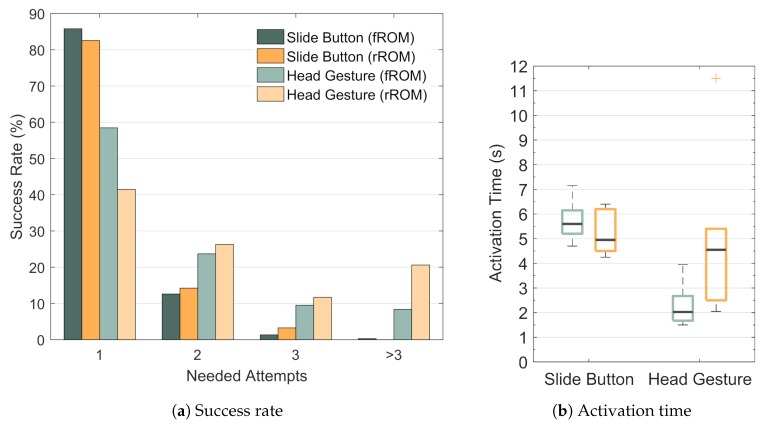
Comparison between Slide Button and Head Gesture. The performance of the Slide Button and the Head Gesture was evaluated in terms of success rate and activation time.

The success rate of the Slide Button was 85.80% for the subjects with full ROM (Figure 12a). For the subjects with restricted ROM it was 82.52%. When the first attempt failed, the subjects with full ROM activated the Slide Button successfully after the second attempt in 12.60% of the cases. The subjects with restricted ROM succeeded after the second attempt in 14.23%. In 1.32%, the subjects with full ROM needed three attempts to successfully activate the Slide Button. The subjects with restricted ROM needed three attempts in 3.25% of the cases. In the remaining 0.28%, more than three attempts were needed by the subjects with full ROM. The subjects with restricted ROM did not need more than three attempts.

In Figure 12b the activation times for the Slide Button and for the Head Gesture are shown based on the median activation times for each subject. For the Slide Button the median activation time was 5.6 s for the subjects with full ROM and 5.0 s for the subjects with restricted ROM. The two-sample t-test [36] indicated no significant difference between both groups. However, for the subjects with full ROM the median activation time of the Head Gesture was 2.0 s. This is almost three times faster than the Slide Button activation, even though all failed activation attempts were included in the activation time. This difference is significant. For the subjects with restricted ROM the median activation time of the Head Gesture was 4.6 s. That means, the subjects with restricted ROM needed significantly more time to perform the Head Gesture correctly than the subjects with full ROM (two-sample t-test). Futhermore, according to a paired-sample t-test, the activation times of the Head Gesture and the Slide Button did not differ significantly for the subjects with restricted ROM.

It is important to note that the execution time for a single Head Gesture had to lie between 0.25 s and 2 s. Movements beyond these bounds were not taken into account by the classification algorithm. That means that a certain execution speed was enforced for the Head Gesture while the Slide Button could be moved as slowly as desired. The fact that the Head Gesture had to be performed both quickly and accurately led to a significant lower success rate of the Head Gesture compared to the Slide Button for both groups (paired-sample t-tests): The success rate of the Head Gesture for the subjects with full ROM was 58.45% and 41.45% for the subjects with restricted ROM. In 23.68% of all cases, the second attempt was successful for the subjects with full ROM. The subjects with restricted ROM succeeded after the second attempt in 26.27% of all cases. Three attempts were needed by the subjects with full ROM in 9.51% of all cases. The subjects with restricted ROM performed the Head Gesture correctly after the third attempt in 11.69% of the cases. In 8.36% of the cases more than three attempts were needed by the subjects with full ROM, while the subjects with restricted ROM needed more than three attempts in 20.59% of the cases. It is notable that the maximum number of observed failed attempts was 16 for the Head Gesture while it was 4 for the Slide Button. Though the non-detection rate of the gesture was relatively high, the robot did not move unintendedly, which is very important from a safety point of view.

In conclusion, for both groups the success rate of the Head Gesture was significantly lower than the one of the Slide Button. On the other hand, the activation time of the Head Gesture was significantly lower for the subjects with full ROM. However, this difference was not present for the subjects with restricted ROM: The results indicate that the correct execution of the Head Gesture becomes more difficult when the ROM is restricted, while based on our results, the level of difficulty of the Slide Button is not affected significantly by the available ROM of the user.

### 4.2. Subjective Evaluation

The Mann-Whitney U-test [40] indicated no significant differences between the subjects with full and restricted ROM. Hence, the ratings from both groups have been assumed to originate from the same distribution and are therefore presented together.

#### 4.2.1. Calibration

As Table 2 shows, the majority of subjects found both the Cursor as well as the Robot Calibration highly intuitive. Furthermore, the acoustic feedback to indicate that a calibration point has been saved was perceived as very useful.

#### 4.2.2. GUI

The subjects considered both the Cursor GUI and the Robot GUI visually appealing and clearly structured. They agreed that all important functions were easily accessible during cursor control. Moreover, the majority of subjects had the opinion that all important information was displayed on the GUI during robot control. A few subjects remarked that more information could be helpful. The feedback showing the current head orientation was experienced as very useful. The camera image was mainly considered useful, even though a few subjects remarked that they had not paid much attention to the camera image during control. This is likely, because it was not mandatory for the given tasks. However, there are scenarios in which the gripper camera is necessary because direct view is obstructed.

#### 4.2.3. Switching

The Slide Button activation was rated very easy. This is also in line with the high success rate of the Slide Button. The vast majority of the subjects perceived the acoustic and visual feedback of the Slide Button as useful or very useful but some subjects found the acoustic feedback disturbing after a while. The Head Gesture has been found to be significantly more difficult than the Slide Button. This is also represented by the lower success rate of the Head Gesture. Some subjects remarked that they perceived the Head Gesture as a stress-inducing factor. Observations during the experiments indicate that the gesture execution was demanding for some subjects with restricted ROM. The entire switching procedure was mainly rated as easy. Most subjects rated switching between groups as too slow. The common opinion was that the Head Gesture and also the Slide Button alone were quick enough but the combination was not. All in all, the Head Gesture was not considered easy to execute and the associated feedback was not considered helpful.

#### 4.2.4. Mapping

In order to evaluate the intuitiveness of the mapping, the subjects were asked how well they could imagine how the robot would respond to their head motion. The results indicate that the subjects found it highly intuitive to open or close the gripper using pitch motion. Furthermore, they strongly agreed that they could well imagine that the robot moved up or down in the Vertical Plane group when they tilted their heads up or down. The majority of the subjects could also imagine well how the robot would move if they rotated their heads left or right. Most subjects agreed that they could imagine well how the robot would move in the Horizontal Plane group when they tilted their heads up or down. However, the vast majority remarked that they would have found it more intuitive if the direction of the robot motion had been switched, that is, that the robot moved closer to the subjects when they tilted their heads down and vice versa. This is also in line with the observation that many subjects initially moved the robot in the wrong direction during movements 4 and 6 of the predefined task. The intuitiveness of the mapping for the DOFs of the Orientation group was significantly lower than for the DOFs of the Vertical Plane group or the Gripper. The subjects criticized that they found it hard to imagine rotations in local coordinates of the gripper. But they agreed that it became a lot easier after they had been instructed to imagine the gripper to have a face with the camera as the eyes and the gripper as the mouth. A possible explanation is that the gripper movements may then be processed in different parts of the brain, such as the Fusiform Face Area. Once the gripper is interpreted as a face, mirror neurons may map its movements onto the subjects’ own head movements.

#### 4.2.5. Transfer Function

**Figure 13:**
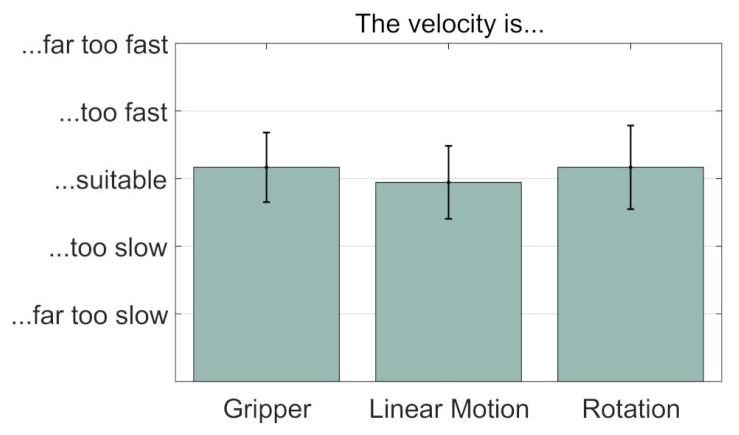
Control speed evaluation. Mean and standard deviation of subjective velocities during gripping, linear motion and rotations (n = 18).

The majority of subjects strongly agreed that it was very easy to move the mouse cursor precisely under the testing conditions. This is in line with the high success rate of the Slide Button. Furthermore, the subjects found precise positioning of the mouse cursor significantly easier than gripping or moving the robot arm precisely. A few subjects annotated that they found the mouse cursor too fast. This problem can be solved by decreasing the distance between subject and screen or by choosing a larger screen. Gripping accurately was mainly considered as easy. Some subjects criticized that the gripper could not be opened and closed smoothly. This problem was caused by the gripper and can only be solved by changing the gripper hardware. On average, subjects agreed that accurate positioning of the robot arm was easy. The subjects who partly agreed to that statement mainly argued that they sometimes lacked feedback for accurate positioning. Nonetheless, all the subjects were able to complete the tasks without colliding. The velocities for opening/closing the gripper, linear motion and rotational motion have been assessed suitable by almost all subjects (Figure 13). More experienced users might find robot control too slow. However, the AMiCUS system allows the adjustment of maximum robot velocity.

#### 4.2.6. General

The majority of subjects found it easy or very easy to assess robot position correctly in both the Vertical Plane and Horizontal Plane group. However, some subjects asked for additional feedback for accurate positioning. Assessing robot orientiation correctly was experienced as significantly more difficult than assessing robot position. Most subjects had no problems with attention loss when looking back and forth between screen, robot and test course. In general, the system has been greatly accepted by the subjects: All of them found that robot control with AMiCUS was fun.

## 5. Conclusions

Within this work, we presented the AMiCUS system and evaluated it in a user study with 13 able-bodied and 6 tetraplegic subjects. In this section, we validate that the requirements are met and we compare the system presented here with previously published work.

### 5.1. Compliance with Requirements for a Head Motion-Based HRI

In the following, we step through the list of criteria for a solely head motion-based HRI in order to validate that all requirements are met:

1. The HRI should be adaptive, always using the full available neck range of motion of the user.

During the calibration routine, AMiCUS is adapted to the available neck range of motion of the user. Possible constraints are only imposed by required head gestures. For our experiments, 6 tetraplegics have been recruited that were unable to operate the AMiCUS version in Reference [29] due to severe head motion limitations that restricted their ability to perform head gestures. All of these subjects were able to successfully operate the robot arm with the version presented here. This makes our version available for a wider range of users. Furthermore, the control structure here can easily be adapted to users that can only use one or two head DOFs by introducing more robot groups with fewer DOFs in the mapping.

2. The relationship between performed head motion and resulting robot motion has to be intuitive.

In general, the relationship between performed head motion and resulting robot motion is intuitive, as all first-time users were able to operate the robot arm. However, further improvements are still possible: The direction of the mapping onto the ${}^{w}z$-axis should be inverted in the future. That means, the robot should move closer to the user when turning the head down and further away when turning the head up. Moreover, performing rotations was generally considered as challenging. This resulted mainly from the fact that subjects found it difficult to imagine rotations in gripper coordinates. However, after they were instructed to imagine the gripper as a face, they perceived rotations as easier. In order to facilitate the interpretation of the gripper as a head, the design of the gripper including the camera may be adapted in a way that the gripper resembles a face more. Further attempts to increase intuitiveness may also focus on performing rotations in world coordinates instead of gripper coordinates. Additionally, future versions of AMiCUS may include an option to disable simultaneous rotations because the vast majority of subjects was confused by coupled rotations. However, observations in our research group strongly indicate that rotations in local coordinates as well as simultaneous rotations are not an issue for more experienced users.

3. The HRI must reliably distinguish between unintended head motion, head motion intended for direct control and head motion to generate switching commands.

It could be demonstrated that the robot did not move unintendedly. That means, AMiCUS could reliably distinguish between head motion intended for robot control and head gestures or unintended head motion. Furthermore, unintended head motion has never been classified as a switching command. These facts give a positive prognosis for safety requirements of a possible product implementation. However, in some cases head motion intended to perform a Head Gesture or Slide Button activation has not been detected because the head movements have not been performed as defined. Given these inevitable imperfections of the user to perform head movements in an accurate and repeatable manner, the control elements and switching process have been analyzed to identify possibilities for improvement:

The Slide Button turned out to be a good control element with both high success and satisfaction rate. In contrast, the success rate of the Head Gesture was significantly lower, resulting in lower user acceptance. Furthermore, motion limitations negatively affect a user’s ability to perform the Head Gesture correctly. The entire switching process was perceived as easy but slow, leaving room for improvement. As the Head Gesture has been perceived as difficult and has been in particular challenging for user’s with restricted ROM, it should be discarded or improved in the future.

4. The HRI has to give sufficient and useful feedback to the user to allow safe and efficient operation.

The results of the study indicate that the calibration procedures are easy to understand and that sufficient and useful feedback is provided. The GUI was generally experienced as visually appealing and clearly structured. Moreover, the subjects agreed that all important functions could be accessed easily and all important information was displayed. A possibility to turn the sound off would be appreciated by most long-term users, though. In some cases, assessing the gripper position along the ${}^{w}z$-axis was perceived as difficult. However, our experience indicates that users will learn to interpret the gripper camera image better after working with AMiCUS for a longer period of time. In conlusion, AMiCUS gives overall sufficient and useful feedback to the user to allow safe and efficient operation.

5. The HRI must enable the user to perform smooth, precise and efficient robot movements in Cartesian space.

Overall, the transfer function was well-adapted to most users during linear motion, rotation and gripping as it enabled accurate positioning while providing a suitable speed of operation. Therefore, AMiCUS enables the user to perform smooth, precise and efficient robot movements. In case the default settings are not suitable, the parameters of the transfer function can be adapted easily to meet the preferences of the user, in particular regarding operation speed.

6. The user should enjoy using the HRI.

User acceptance is often a critical point for assistive devices, jeopardizing their success as a product. AMiCUS, in contrast, received high user acceptance since the subjects of this study considered robot control with AMiCUS as fun.

In summary, AMiCUS fulfills all the requirements of a head-controlled HRI for robot arm control and has been greatly accepted by the subjects.

### 5.2. Comparison with Previously Published Work

When using the interface presented here, the user has to perform a Head Gesture and then activate a Slide Button in order to switch between robot groups. Even though the switching process can still be improved, switching will inherently take longer than for the interface presented by Jackowski et al. [29], which uses each one out of four different gestures to switch between robot groups. This direct group access makes switching very quick. However, the correspondence between gestures and robot groups depends on the current robot group, which is a general source of confusion and sometimes makes users select the wrong group. Furthermore, performing head gestures is complex and involves many muscles, some of which might not be available for tetraplegics with severe head motion limitations. As a matter of fact, out of 13 randomly picked tetraplegics, only 6 were capable to use the gesture-based interface presented by Jackowski et al. This strongly limits the applicability of this interface for individuals with head motion restrictions. Even more, if the restrictions are such that one or two head DOFs cannot be used for control: The interface presented within this work offers the possibility to change the mapping as described previously. This is hardly possible for the interface presented in Reference [29] because each new group resulting from a lost head DOF requires a new head gesture, whereas the ability to produce head gestures decreases with increasing head motion restrictions. For these reasons, we conclude that the interface presented by Jackowski is promising as a hands-free interface for people without head motion limitations, whereas the interface presented here is more suitable for individuals with head motion restrictions because it can be adapted to their special needs.

## Figures and Tables

**Table 1:** Settings of the Hillcrest FSM-9.

Setting	Value	Meaning
Samplerate	125Hz	
Operating Mode	4	Full Motion on
Packet Select	8	Motion Engine output
Format Select	0	Format 0 packet
FF2	true	Enable output of linear acceleration, no gravity
FF6	true	Enable output of angular position

**Table 2:** Evaluation sheet for AMiCUS including results.

Topic	No.	Statement	Rating
**Calibration**	1	Understanding what one is supposed to do during Cursor Calibration is easy	4.83±0.38
	2	Understanding what one is supposed to do during Robot Calibration is easy	4.81±0.40
	3	The acoustic feedback during calibration is useful	4.88±0.33
**GUI**	4	The Cursor GUI is visually appealing and clearly structured	4.39±0.61
	5	In Cursor Mode, all important functions can be accessed easily	4.61±0.61
	6	The Robot GUI is visually appealing and clearly structured	4.33±0.69
	7	In Robot Mode, all important information is displayed on the GUI	4.41±0.71
	8	The feedback about the current head position is easy to understand and useful	4.78±0.43
	9	The camera image is useful	4.33±1.14
**Switching**	10	Activating the Slide Button is easy	4.56±0.62
	11	The acoustic and visual feedback for the Slide Button is useful	4.44±0.92
	12	Performing the Head Gesture correctly is easy	3.33±1.46
	13	The feedback about Head Gesture execution is useful	3.89±1.37
	14	Switching between groups is easy	4.06±0.94
	15	Switching between groups is quick	3.33±0.91
**Mapping**	16	I can well imagine what the gripper does when I move my head up or down	4.61±0.78
	17	I can well imagine what the robot does in Vertical Plane group when I move my head up or down	4.72±0.75
	18	I can well imagine what the robot does in Vertical Plane or Horizontal Plane group when I turn my head to the left or to the right	4.56±0.86
	19	I can well imagine what the robot does in Horizontal Plane group when I move my head up or down	4.28±1.02
	20	I can well imagine what the robot does in Orientation group when I move my head up or down	3.44±1.25
	21	I can well imagine what the robot does in Orientation group when I turn my head to the left or to the right	3.44±1.15
	22	I can well imagine what the robot does in Orientation group when I bend my head to the left or to the right	3.33±1.24
**Transfer function**	23	Moving the mouse cursor precisely is easy	4.44±0.62
	24	Gripping precisely is easy	4.17±1.15
	25	Moving the robot arm precisely is easy	4.44±0.70
**General**	26	Assessing robot position correctly in Vertical Plane group is easy	4.56±0.70
	27	Assessing robot position correctly in Horizontal Plane group is easy	4.28±0.75
	28	Assessing robot orientation correctly is easy	2.89±1.18
	29	I can easily keep an eye an all relevant parts of the system	3.94±1.26
	30	Robot control is fun	4.72±0.46

Rating: 1 = “I strongly disagree”, 2 = “I disagree”, 3 = “I partly agree”, 4 = “I agree”, 5 = “I strongly agree”.

## Supplementary Materials

The following are available online at https://www.mdpi.com/1424-8220/19/12/2836/s1, Static Data S1: 5 min of static data recorded with Hillcrest FSM-9, Dynamic Data S1: Head motion data recorded with Hillcrest FSM-9.

## Abbreviations

The following abbreviations are used in this manuscript:

AHRS	Attitude Heading Reference System
AMiCUS	Adaptive Head Motion Control for User–friendly Support
DOF	Degree of Freedom
GUI	Graphical User Interface
HMI	Human–Machine Interface
HRI	Human–Robot Interface
MEMS	Micro Electro-Mechanical System
ROM	Range of Motion
TCP	Tool–Center Point
